# Exploring focal adhesion data: dynamic parameter extraction from FRAP and FLAP experiments using chemical master equation

**DOI:** 10.3389/fmolb.2025.1587608

**Published:** 2025-05-06

**Authors:** Luciana Renata de Oliveira, Matheus Gimenez Fernandes, José Salvatore Leister Patane, Jean-Marc Schwartz, José Eduardo Krieger, Christoph Ballestrem, Ayumi Aurea Miyakawa

**Affiliations:** ^1^ Laboratório de Genetica e Cardiologia Molecular, Instituto do Coração (InCor), Hospital das Clinicas HCFMUSP, Faculdade de Medicina, Universidade de Sao Paulo, Sao Paulo, Brazil; ^2^ School of Biological Sciences, Faculty of Biology, Medicine and Health, University of Manchester, Manchester, United Kingdom; ^3^ Manchester Cell-Matrix Centre, Division of Cell-Matrix Biology and Regenerative Medicine, School of Biological Sciences, Faculty of Biology, Medicine and Health, University of Manchester, Manchester, United Kingdom

**Keywords:** chemical master equation, focal adhesion, FRAP, FLAP, protein dynamics, protein interaction

## Abstract

The dynamic behavior of proteins within cellular structures can be studied using fluorescence recovery after photobleaching (FRAP) and fluorescence loss after photobleaching (FLAP) experiments. These techniques provide insights into molecular mobility by estimating parameters such as turnover rates $\left(k_{T}\right)$ and diffusion coefficients (D). However, traditional deterministic models often rely on simplifying assumptions that may not fully capture the stochastic nature of molecular interactions. In this study, we developed a novel stochastic model based on the analytical solution of the chemical master equation to extract dynamic parameters from FRAP and FLAP experiments in the focal adhesion (FA) network. Our approach extends beyond standard FRAP/FLAP analysis by inferring additional parameters, such as protein-specific entry $\left(k_{In}\right)$ and exit $\left(k_{\mathrm{Out}}\right)$ rates, allowing a deeper understanding of protein turnover and interactions. To validate our model, we analyzed previously published experimental data from NIH3T3 fibroblasts expressing GFP-tagged FA proteins, including tensin 1, talin, vinculin, α -actinin, ILK, α -parvin, kindlin-2, paxillin, p130Cas, VASP, FAK, and zyxin. These proteins participate in mechanotransduction, cytoskeletal organization, and adhesion regulation, exhibiting distinct dynamic behaviors within FA structures. Furthermore, we constructed an interaction network to quantify how vinculin and actin influence talin dynamics, leveraging our model to uncover their regulatory roles in FA turnover. Using an analytical solution of the chemical master equation, our framework provides a generalizable approach for studying protein dynamics in any system where FRAP and FLAP data are available. It can be applied to new experimental datasets and reanalyzed from existing data, revealing previously inaccessible molecular interactions and enhancing our understanding of FA dynamics and broader cellular processes.

## 1 Introduction

Fluorescence recovery after photobleaching (FRAP) and fluorescence loss after photobleaching (FLAP) are two related imaging techniques used in cell biology to study the dynamics of fluorescently labeled proteins within living cells. These techniques provide valuable insights into the movement, interactions, and turnover rates of cellular components (Day et al., 2012; Dunn et al., 2002). In FRAP, a specific region of interest within a cell is selected to study a labeled molecule with a fluorescent marker, typically a fluorescently tagged protein or lipid. This region is subjected to intense light, such as from a laser, which bleaches the fluorophores in that area, rendering them non-fluorescent. The fluorescence recovery in the bleached area over time is then monitored using a fluorescence microscope. As fluorescent molecules from the surrounding unbleached areas diffuse into the bleached region, the fluorescence signal gradually returns, allowing researchers to measure the rate and extent of recovery. This recovery can provide valuable information about the labeled molecule’s mobility, turnover, and interactions within the cell (Sprague and McNally, 2005; Lippincott-Schwartz et al., 2001; Axelrod et al., 1976; Hickey et al., 2021). FLAP, on the other hand, is focused on studying the mobility and dynamics of specific proteins within living cells. The molecule to be located carries two fluorophores: one to be photobleached and the other to act as a reference label. The use of a reference fluorophore permits the distribution of the photo-labeled molecules themselves to be tracked by simple image differencing. FLAP is comparable with methods to track fluorescent proteins by direct photoactivation, however, instead of monitoring the overall recovery of fluorescence within the bleached area alike in FRAP, it involves tracking the movement of fluorescently labeled molecules into and out of subcellular structures. This allows researchers to assess not only the overall mobility of the labeled molecules but also their specific localization within different cellular compartments (Lippincott-Schwartz et al., 2001; Hickey et al., 2021).

Mathematical modeling of FRAP and FLAP data allows the determination of dissociation and association rates ( $k_{\mathrm{Off}}$ and $k_{On}$ ), distribution of mobile and immobile fractions, and corresponding diffusion coefficients (D) (Lippincott-Schwartz et al., 2018; Giakoumakis et al., 2017; Carrero et al., 2003; Mai et al., 2011). These data can be analyzed by employing deterministic or stochastic mathematical models. Deterministic models assume that molecular behavior is predictable and use differential equations to describe the evolution of the concentration or intensity of the fluorescent molecules over time and space (Ellenberg et al., 1997; Houtsmuller et al., 1999; Braeckmans et al., 2003; Braga et al., 2004; Mazza et al., 2008). In contrast, stochastic models consider the random nature of molecular movements and interactions and the variability and noise in the experimental data. In this way, stochastic models can provide a more accurate and realistic estimation of the diffusion and binding parameters than deterministic models (Blumenthal et al., 2015; Moraru et al., 2008; Bläßle et al., 2018; Röding et al., 2019; Nicolau et al., 2007; Groeneweg et al., 2014; Carnell et al., 2015; Dallon et al., 2022; Riznichenko and Rubin, 2021; Vilaseca et al., 2011).

Existing models analyzing FRAP and FLAP experiments are often deterministic and rely on simplification and assumptions about particular parameter values to calculate the analytical solution (Phair et al., 2003; Phair and Misteli, 2001; Lippincott-Schwartz et al., 2018). In this work, we are proposing the first stochastic mathematical model that relies on only 2 parameters to explain the dynamics of protein behavior inside the FA (region of interest - R.O.I.). The model is very straightforward and does not require any prior assumptions. It uses only the information of the turnover rate $\left(k_{T}\right)$ and the stationary concentration of mobile proteins ( $\bar{n_{in}^{P}}$ ), that are traditionally extracted from FRAP and FLAP experiments.

**FIGURE 1:**
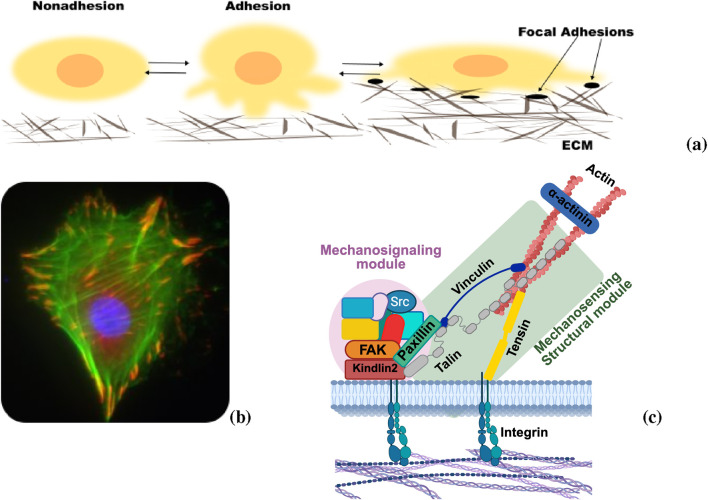
Integrin-mediated cell-matrix adhesions. **(a)** During cell spreading, cells form cell-matrix adhesions, which in cultured cells are called Focal Adhesions (FAs). **(b)** Fluorescence microscopy of. Smooth muscle cell stained for the FA marker paxillin (red) and filamentous actin (F-actin; green); nucleus in blue (image captured by the authors). **(c)** Model of molecular constituents of FA proposed by (Stutchbury et al., 2017). The model displays two modules involved in mechanotransduction: the mechanosensing module, comprising proteins that form a direct link to the contractile actomyosin (e.g., talin and vinculin), and other regulatory proteins that are involved in signaling processes (kindlin, FAK, paxillin and other proteins) (Created in BioRender. DE OLIVEIRA, L. (2025) https://BioRender.com/2q1onya).

Our study examines FRAP and FLAP outcomes for 12 proteins within the focal adhesion (FA). FA are multiprotein assemblies that manifest as localized adhesive structures, readily observable through fluorescence microscopy, as illustrated in Figure 1. More than 2,000 proteins have been associated with a wider protein network of FAs, and about 60 of them are core adhesion proteins that play a direct role in regulating cell-matrix adhesion (Atherton et al., 2015; 2016; Jansen et al., 2017; Kanchanawong et al., 2010). Based on their dynamic turnover rates, these proteins can be distinctly categorized into mechanosignaling, intermediate and mechanosensing modules, as proposed by (Stutchbury et al., 2017) and illustrated in Figure 1. Mechanosensing proteins are those that link integrins to the contractile force machinery and mechanosignalling proteins modulate this link through signals that control Rho GTPases which in turn influence the actin polymerization or/and actomyosin contractility. Despite the advances in our understanding, the mechanisms by which the different proteins cooperate and coordinate the communication of the cells with the surrounding extracellular matrix (ECM) are still unclear.

Here, we introduce a stochastic model grounded in the analytical solution of the chemical master equation (CME), which we apply to analyze FRAP and FLAP data. Our model estimates the rates of protein entrance $\left(k_{In}\right)$ and exit $\left(k_{\mathrm{Out}}\right)$ from the focal adhesion region of interest (ROI), providing deeper insights into protein dynamics compared to traditional turnover rates. Furthermore, our model predicts how perturbations, such as mutations that disrupt protein interactions, might affect protein behavior. This approach opens new avenues for *in silico* testing of protein interactions and provides valuable experimental insights into FA dynamics and other cellular processes.

## 2 Methods

### 2.1 Experimental data

**FIGURE 2:**
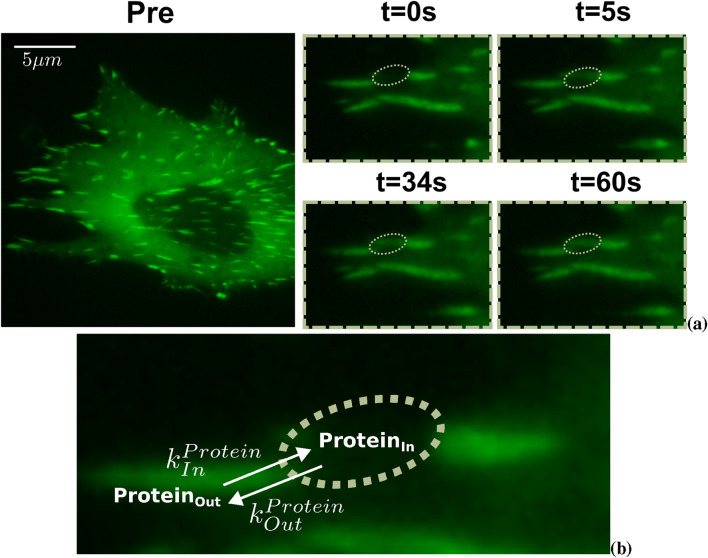
Experimental and theoretical framework for studying focal adhesion protein dynamics. **(a)** Example of time-lapse images from a Fluorescence Recovery After Photobleaching (FRAP) experiment in NIH3T3 fibroblasts transfected with a GFP-tagged FA protein. The dashed box indicates the region of interest (R.O.I.) selected for photobleaching, corresponding to a FA. Insets show magnified views of the R.O.I. at different time points post-bleaching, highlighting fluorescence recovery within the bleached region (dotted ellipse). **(b)** Schematic representation of the abstract model used for interpreting FRAP and Fluorescence Loss After Photobleaching (FLAP) experiments. Proteins outside the FA region (Protein_Out_) can enter (rate $k_{\text{In}}^{\text{Protein}}$ ) and proteins inside (Protein_In_) exit (rate $k_{\text{Out}}^{\text{Protein}}$ ) the FA region. The green area represents the experimental R.O.I., corresponding to a focal adhesion.

The experimental data were obtained from previous FRAP and FLAP data sets. For FRAP experiments, NIH3T3 fibroblasts were transfected with GPF-tagged FA proteins of interest (tensin 1, talin, vinculin, a-actinin, ILK, a-parvin, kindlin-2, paxillin, p130Cas, VASP, FAK or zyxin, Figure 2a). Photobleaching was achieved with a 488 nm laser and fluorescence recovery monitoring at 10-s intervals for up to 5 min using a DeltaVision system RT microscope (Stutchbury et al., 2017).

For FLAP experiments, NIH 3T3 cells were transfected with PAGFP-tagged protein of interest (talin-full length, talinΔR1R10, talinΔR2R3 or talinΔR4R10) and mCherry-tagged marker. Photoactivation was performed with a 405 nm laser and imaging was conducted using a spinning disk confocal microscope (Atherton et al., 2015).

### 2.2 Extracting dynamic parameters from FRAP and FLAP experiments

FRAP and FLAP experimental data consist of fluorescence intensity curves representing the recovery or the loss of intensity, respectively (Ishikawa-Ankerhold et al., 2012). These curves have an exponential shape and can be fitted using the equation $y=\;y_{0}+Ae^{R_{0}x}$ , thus yielding values for the parameters $y_{0}$ , A , and $R_{0}$ (see Supplementary Figure S1).

The analytical solution of the chemical master equation used in our model relies on two experimental parameters: the turnover rate $\left(k_{T}\right)$ and the stationary concentration of mobile proteins $\left(\bar{n_{In}^{P}}\right)$ . The turnover rate is obtained from the fit of the experimental fluorescence recovery data and is calculated as $k_{T}={|R}_{0}|$ , representing the protein’s mobility in the evaluated experiment. The stationary concentration of mobile proteins $\left(\bar{n_{In}^{P}}\right)$ is inferred from the fluorophore intensity at the last time point of each experiment, which is considered to represent the stationary population of the mobile protein within the region of interest (ROI). In our model, the chemical master equation describes the time evolution of the protein population, and it is assumed that the fluorescence intensity is proportional to the number of molecules in the system, as commonly adopted in fluorescence-based quantitative studies (Elowitz et al., 2002; Zhang et al., 2016; Muñoz-Cobo and Berna, 2019; Qian and Bishop, 2010). This definition ensures that our model directly incorporates experimentally measured values to describe protein dynamics.

### 2.3 Stochastic model of master equation

The stochastic mathematical model is built using a combination of experimental data from FRAP and FLAP (Atherton et al., 2015; Stutchbury et al., 2017) and the formalism of the chemical master equation (De Oliveira, 2014; Qian and Bishop, 2010; Van Kampen, 1992). The chemical master equation is a class of discrete-state, continuous-time Markov jump processes, known as multi-dimensional birth-death processes in probability theory (Pinsky and Karlin, 2010; Van Kampen, 1992). In this formalism, the concentration of proteins is modeled as temporal variables assuming non-negative real values. These processes are continuous in time, their range consists of integers, and only jumps between adjacent states are permitted (Van Kampen, 1992).

#### 2.3.1 Protein in-out model

Based on the experimental data, we propose a model to describe protein dynamics in FRAP/FLAP experiments. In this model, the protein population is represented by its concentration inside $\left(n_{In}^{P}\right)$ and outside $\left(n_{\mathrm{Out}}^{P}\right)$ the FA (Figure 2b). The temporal evolution of these populations is governed by the transition rates $k_{In}^{\mathrm{Protein}}$ and $k_{\mathrm{Out}}^{\mathrm{Protein}}$ , which define the protein flux into and out of the FA over time. Given the time scales of the experiments (Atherton et al., 2016; Stutchbury et al., 2017), the total protein concentration can be considered constant:

$$n_{\mathrm{Tot}}^{P}=n_{In}^{P}+n_{\mathrm{Out}}^{P}.$$ (1)

This means that the system is fully described in terms of one of the two populations, $n_{In}^{P}$ or $n_{\mathrm{Out}}^{P}$ and we choose to write our model in terms of the proteins inside $\left(n_{In}^{P}\right)$ the FA (R.O.I.).

#### 2.3.2 Inference of dynamic parameter using the analytical solution of the chemical master equation

Following the Protein-In-Out model 2.3.1, the one protein FRAP/FLAP experiment is fully described by a one-dimensional chemical master equation (Van Kampen, 1992), that is written as:

$$\begin{array}{ll} \frac{dp\left(n_{In}^{P}\left(t\right)\right)}{dt} & =r\left(n_{In}^{P}\left(t\right)+1\right)p\left(n_{In}^{P}\left(t\right)+1\right)+g\left(n_{In}^{P}\left(t\right)-1\right)\\ & \;p\left(n_{In}^{P}\left(t\right)-1\right)-\left(r\left(n_{In}^{P}\left(t\right)\right)+g\left(n_{In}^{P}\left(t\right)\right)p\left(n_{In}^{P}\left(t\right)\right)\right. \end{array}$$ (2)

Where the protein concentration over time inside the FA is described by the probability $p\left(n_{In}^{P}\left(t\right)\right)$ of finding the system in that state. The time evolution of $p\left(n_{In}^{P}\left(t\right)\right)$ is represented by a combination of:

1. The gain term, $g\left(n_{In}^{P}\left(t\right)\right)=k_{In}^{\mathrm{Protein}}n_{\mathrm{Out}}^{P}\left(t\right)$ , responsible for the increment of the concentration of the protein inside the FA;
2. The recombination term, $r\left(n_{In}^{P}\left(t\right)\right)=k_{\mathrm{Out}}^{\mathrm{Protein}}n_{In}^{P}\left(t\right)$ , responsible for the reduction of the concentration of the protein inside the FA.

The system represented by Equation 2 respects the detailed balance condition, which means the system has an exact analytical solution (Toral and Colet, 2014),

$$\begin{array}{cc} n_{In}^{P}\left(t\right)= & n_{In}^{P}\left(t_{0}\right)\frac{k_{\mathrm{Out}}^{\mathrm{Protein}}+k_{In}^{\mathrm{Protein}}\exp\left(-\left(k_{\mathrm{Out}}^{\mathrm{Protein}}+k_{In}^{\mathrm{Protein}}\right)\left(t-t_{0}\right)\right)}{k_{\mathrm{Out}}^{\mathrm{Protein}}+k_{In}^{\mathrm{Protein}}}\\ & +n_{\mathrm{Out}}^{P}\left(t_{0}\right)\frac{k_{\mathrm{Out}}^{\mathrm{Protein}}\left(1-\exp\left(-\left(k_{\mathrm{Out}}^{\mathrm{Protein}}+k_{In}^{\mathrm{Protein}}\right)\left(t-t_{0}\right)\right)\right)}{k_{\mathrm{Out}}^{\mathrm{Protein}}+k_{In}^{\mathrm{Protein}}} \end{array}$$ (3)

Where $n_{In}^{P}\left(t_{0}\right)$ and $n_{\mathrm{Out}}^{P}\left(t_{0}\right)$ are the initial concentration of proteins inside and outside the FA, respectively.

From Equation 3, the concentration of proteins in the stationary state $\bar{n_{In}^{P}}$ in terms of $k_{In}^{\mathrm{Protein}}$ and $k_{\mathrm{Out}}^{\mathrm{Protein}}$ is written as follows:

$$\bar{n_{In}^{P}}=\frac{k_{\mathrm{Out}}^{\mathrm{Protein}}}{k_{\mathrm{Out}}^{\mathrm{Protein}}+k_{In}^{\mathrm{Protein}}}$$ (4)

The turnover rate of FRAP/FLAP experiments is represented as the combination of $k_{In}^{\mathrm{Protein}}$ and $k_{\mathrm{Out}}^{\mathrm{Protein}}$ (Sprague et al., 2004; Sprague and McNally, 2005),

$$k_{T}=k_{\mathrm{Out}}^{\mathrm{Protein}}+k_{In}^{\mathrm{Protein}}$$ (5)

With Equations 4, 5 the experimental values of $k_{In}^{\mathrm{Protein}}$ and $k_{\mathrm{Out}}^{\mathrm{Protein}}$ are determined as:

$$\left\{\begin{array}{c} k_{\mathrm{Out}}^{\mathrm{Protein}}=\bar{n_{In}^{P}}\cdot k_{T}\;\\ k_{In}^{\mathrm{Protein}}=k_{T}-k_{\mathrm{Out}}^{\mathrm{Protein}}\; \end{array}\right.$$ (6)

Where the stationary concentration of mobile proteins $\left(\bar{n_{In}^{P}}\right)$ and the turnover rate $\left(k_{T}\right)$ are extracted directly from fitting the FRAP experiments (Lippincott-Schwartz et al., 2018; Blumenthal et al., 2015; Giakoumakis et al., 2017; Sprague and McNally, 2005) (Section 2.2). The results are shown as normalized data in function of the total intensity per protein. In the case of FLAP, the stationary concentration of mobile proteins inferred is $\bar{n_{\mathrm{Out}}^{P}}$ , which can similarly be used following Equation 1.

#### 2.3.3 Inference of protein interaction using the chemical master equation

**FIGURE 3:**
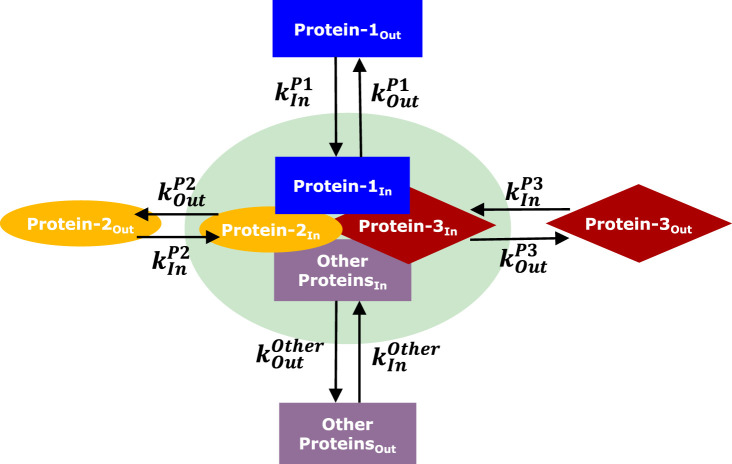
Representation of FA protein interactions considering 3 proteins to be studied, where only the interactions of protein 2 and 3 with protein 1 are considered. The model considers the entrance and exit of proteins in and out of the FA (green region). Each protein has one pair of dynamic rates $k_{In}^{P_{n}}$ and $k_{\mathrm{Out}}^{P_{n}}$ , where n is the protein is the protein identification ( $Protein_{1}$ , $Protein_{2}$ or $Protein_{3}$ ).

Data from individual protein experiments (Section 2.3.1, Section 2.3.2) was used to build a network representing the interaction of proteins inside the FA. At this step, the dynamic interaction between proteins was described by the chemical master equation divided into two steps: 1) the entrance and exit of proteins in the FA, and 2) the interaction of those proteins (Figure 3). FA is a complex of more than 60 proteins (Jansen et al., 2017; Kanchanawong et al., 2010; Zaidel-Bar et al., 2003), and the experimental data measure a combinatory behavior of the interaction of the protein with some other proteins in the FA. Figure 3 is a simplification of this system considering 3 proteins, where all other proteins of the FA complex are taken into account by the term “other proteins”.

In order to understand the influence of $Protein_{2}$ and $Protein_{3}$ , on $Protein_{1}$ dynamics, the chemical master equation (Equation 2) is represented by the following terms (De Oliveira, 2014; Qian and Bishop, 2010; Van Kampen, 1992):

$$\left\{\begin{array}{c} g\left(n_{In}^{P_{1}}\left(t\right)\right)=k_{In}^{P_{1}}{\cdot n}_{\mathrm{Out}}^{P_{1}}\left(t\right)+\left(k_{In}^{P_{2}}+k_{In}^{P_{3}}\right){\cdot n}_{In}^{P_{1}}\left(t\right)\;\\ r\left(n_{In}^{P_{1}},t\right)=\left(k_{\mathrm{Out}}^{P_{1}}{\cdot n}_{In}^{P_{1}}\right)\; \end{array}\right.$$ (7)

Where ${g(n}_{In}^{P_{1}}\left(t\right))$ is considering the entrance of $Protein_{1}$ in the FA, $k_{In}^{P_{1}}$ , and the recruitment of proteins $Protein_{2}$ , $k_{In}^{P_{2}}$ and $Protein_{3}$ , $k_{In}^{P_{3}}$ and their interaction with $Protein_{1}$ . The term ${r(n}_{In}^{P_{1}}\left(t\right))$ is representing the exit of $Protein_{1}$ from the FA. As Equation 7 does not have an analytical solution, the Gillespie algorithm (Gillespie, 1977; Liang and Qian, 2010) was used as a numerical solution to solve the system.

### 2.4 Statistical methods

#### 2.4.1 Outliers removal

The presence of outliers in the experimental data was examined, presenting a challenge due to the experiments having a bi-dimensional nature (temporal curves) rather than one-dimensional points. To address this, each curve of each protein was individually fitted, resulting in a unique $R_{0}$ parameter associated with its mobility.

Outliers were identified from the $R_{0}$ values of each protein using the interquartile range (IQR) method (Equation 8), defined as follows:

$$IQR\;=Q_{3}-Q_{1}$$ (8)

Also the definition of the upper (UCL) and lower (LCL) limits, as follows (Equation 9):

$$\left\{\begin{array}{c} UCL\;=Q_{3}+1.5IQR\;\\ LCL\;=Q_{1}+1.5IQR\; \end{array}\right.$$ (9)

After determining UCL and LCL , we excluded from our sample space the curves whose $R_{0}$ values were considered outliers. To compute $k_{T}$ for each protein, we first obtained an average experimental curve for each protein and then applied the fitting procedure described in Section 2.2 to determine $k_{T}$ and $\bar{n_{In}^{P}}$ . The use of average curves is justified by the fact that the model does not account for the parameters $y_{0}$ and A , which can cause shifts in the curves.

#### 2.4.2 Cross-validation

As the experimental data used to calculate the dynamic parameters $k_{In}$ and $k_{\mathrm{Out}}$ was the same used to compare with the analytical curve (Section 2.3.2), there is a risk of introducing overfitting. Although the model accurately represents the existing dataset, its predictive capacity for new experimental replicates remains uncertain. To assess the model’s predictive performance and mitigate the risk of overfitting during parameter calculation, *K-Fold* cross-validation was employed.

##### 2.4.2.1 *K-Fold* cross-validation

*K-Fold* cross-validation (Borra and Di Ciaccio, 2010) is a technique that partitions the dataset into K subsets. Each iteration of this method involves training the model K times, with each subset serving as an independent validation set to calculate the prediction error. This process yields K estimates of the model’s predictive performance. To qualitatively interpret the predictive capacity of the model, the Mean Absolute Percentage Error (MAPE) was used Table 1 (Lewis, 1982). A MAPE value below 20% indicates that the model is suitable for the prediction of the protein behavior.

**TABLE 1:** MAPE interpretability (Lewis, 1982).

MAPE (%)	Interpretation
<10	Highly accurate forecasting
10–20	Good forecasting
20–50	Reasonable forecasting
>50	Inaccurate forecasting

## 3 Results

In this section, we present the results of our analysis using the analytical solution of the chemical master equation applied to FRAP and FLAP data. The experimental data used in this study were extracted from previously published datasets of FRAP and FLAP experiments (Stutchbury et al., 2017; Atherton et al., 2015). The dynamic parameters, turnover rate $\left(k_{T}\right)$ and stationary concentration $\left(\bar{n_{in}^{P}}\right)$ , were calculated (Section 2.2; Supplementary Section 1.2). To ensure the robustness of our results, outliers were excluded from the analysis, and the number of independent experiments used to determine the dynamic parameters for each protein is summarized in Supplementary Table S1. Although the outliers may contain important data, their removal was intended to filter out adverse effects of the experimental procedure, obtaining a more regular pattern in each protein’s behavior and an easier-to-reproduce value of the subsequently calculated parameters (such as $k_{T},k_{In}$ and $k_{\mathrm{Out}}$ ).

The analysis of the dynamic parameters was performed in several steps: first, we evaluated the performance prediction of the analytical solution of the chemical master equation, followed by the determination of the dynamic parameters. Next, we explored the influence of $k_{In}$ and $k_{\mathrm{Out}}$ on the dynamics of focal adhesion (FA) proteins, which allowed us to investigate protein interactions in the context of the FA network.

Our findings demonstrate that the model effectively captures the dynamics of proteins within the FA, offering insights into protein turnover and interactions that go beyond the traditional analysis of $k_{T}$ . By using $k_{In}$ and $k_{\mathrm{Out}}$ , we were able to quantify the influence of specific protein interactions and infer how these interactions shape protein behavior within the FA.

### 3.1 Performance evaluation of the analytical solution to the chemical master equation using K-Fold cross-validation

The K-Fold cross-validation was performed on the outlier-free dataset. The choice of K represents a trade-off between bias and variance (Borra and Di Ciaccio, 2010), therefore the data was cross-validated using K = 3, K = 5 and K = 10. For each value of K the cross-validation procedure was reproduced 100 times. The MAPE obtained across all K values fall within the “Highly accurate” to “Good” ranges, as defined by Table 2. Therefore, the model possesses strong predictive capabilities of protein dynamic behavior by the analytical solution. The model’s validity is maintained even when outlier data is retained in the dataset (Supplementary Table S3).

**TABLE 2:** *K-Fold* cross-validation results: The results are interpreted following Table 1. For the three studied values of K (3, 5 and 10), 100 reproductions were performed, the averages of MAPE values are presented with their respective uncertainties.

Protein	K = 3		K = 5		K = 10	
	MAPE(%)	Interpretation	MAPE(%)	Interpretation	MAPE(%)	Interpretation
Tensin1	7.25±0.10	Highly accurate	7.61±0.09	Highly accurate	8.88±0.10	Highly accurate
Talin1	11.5±0.4	Good	13.2±0.4	Good	17.9±0.4	Good
Vinculin	6.63±0.18	Highly accurate	7.45±0.16	Highly accurate	9.18±0.15	Highly accurate
α -Actinin	7.05±0.15	Highly accurate	7.48±0.12	Highly accurate	9.18±0.15	Highly accurate
ILK	7.62±0.19	Highly accurate	8.44±0.19	Highly accurate	10.25±0.17	Good
α -Parvin	7.52±0.17	Highly accurate	8.32±0.15	Highly accurate	9.70±0.13	Highly accurate
Kindlin2	8.59±0.25	Highly accurate	9.83±0.24	Highly accurate	11.99±0.24	Good
Paxillin	9.80±0.28	Highly accurate	10.72±0.29	Good	14.78±0.33	Good
p130Cas	7.53±0.21	Highly accurate	8.89±0.23	Highly accurate	11.26±0.18	Good
VASP	8.79±0.33	Highly accurate	11.06±0.34	Good	14.62±0.27	Good
FAK	6.98±0.24	Highly accurate	8.10±0.20	Highly accurate	10.93±0.20	Good
Zyxin	5.37±0.14	Highly accurate	6.22±0.12	Highly accurate	7.59±0.11	Highly accurate

### 3.2 Determination of dynamic parameters through the analytical solution using the chemical master equation

The analytical solution of the chemical master equation (see Section 2.3.2) demonstrated strong predictive performance in modeling protein dynamics, as indicated in Table 2. The dynamic parameters $k_{In}$ and $k_{\mathrm{Out}}$ (Table 3) were calculated using Equation 6, based on experimental FRAP data. This data includes the turnover rate $\left(k_{T}\right)$ , the steady-state protein concentration $\left(\bar{n_{in}^{P}}\right)$ , and the percentage of fluorescence intensity observed in FRAP (see Supplementary Section 1.2 for details). These parameters were then used to generate dynamic curves representing protein behavior, with each curve corresponding to one of the 12 FA proteins.

**TABLE 3:** Dynamic rates of FA proteins: The turnover rate $k_{T}$ and the protein concentration in the steady state inside the focal adhesion (Equation 4) were extracted directly from the exponential fit of the intensity distribution of FRAP data. The dynamic parameters $k_{In}$ and $k_{\mathrm{Out}}$ were calculated with the analytical solution in Equation 6.

Protein	$k_{T}$ ( $10^{-3}$ /s)	$k_{In}$ ( $10^{-3}$ /s)	$k_{\mathrm{Out}}$ ( $10^{-3}$ /s)	$\bar{n_{in}^{P}}$ (normalized intensity)
Tensin1	11.29 ± 0.32	4.94 ± 0.20	6.35 ± 0.23	0.562 ± 0.013
Talin1	15.0 ± 0.6	7.8 ± 0.5	7.2 ± 0.5	0.481 ± 0.030
Vinculin	17.7 ± 0.7	5.2 ± 0.4	12.5 ± 0.6	0.706 ± 0.021
α− Actinin	25.7 ± 1.4	6.1 ± 0.6	19.6 ± 1.2	0.761 ± 0.020
ILK	23.4 ± 1.2	9.3 ± 0.6	14.1 ± 0.8	0.603 ± 0.017
α− Parvin	26.1 ± 1.5	10.7 ± 0.8	15.4 ± 1.0	0.590 ± 0.018
Kindlin2	28.8 ± 1.8	10.6 ± 0.9	18.1 ± 1.3	0.630 ± 0.022
Paxillin	37.3 ± 2.5	14.2 ± 1.5	23.1 ± 1.9	0.619 ± 0.031
p130Cas	45.0 ± 3.1	12.5 ± 1.4	32.6 ± 2.5	0.724 ± 0.026
VASP	86 ± 7	19.2 ± 3.4	66 ± 6	0.78 ± 0.04
FAK	83 ± 6	29.1 ± 3.0	54 ± 5	0.649 ± 0.024
Zyxin	87 ± 6	14.9 ± 2.2	72 ± 6	0.829 ± 0.021

**FIGURE 4:**
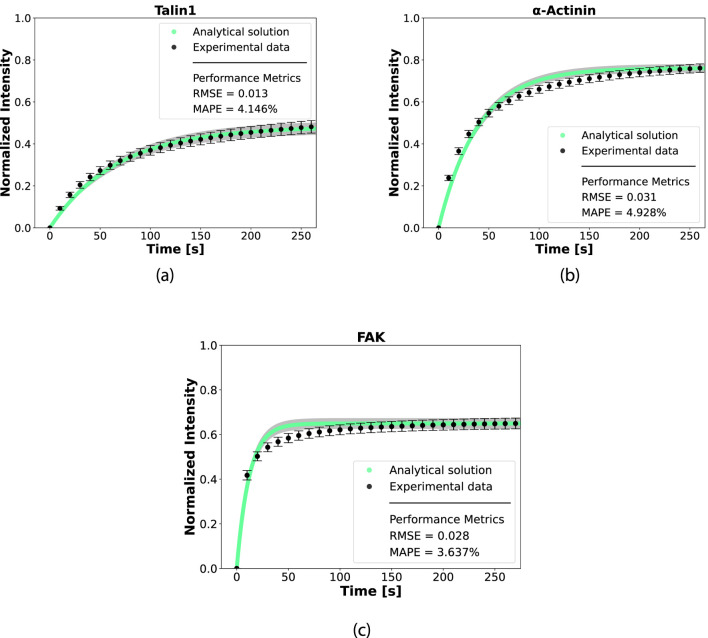
Performance of the model Protein In-Out model using the analytical solution of the chemical master equation for three proteins of each mechanotransduction modules. **(a)** mechanosensing module: Talin1; **(b)** intermediate module: α -actinin; **(c)** mechanosignaling module: FAK. Analytical curves are represented in green ( • ) with the uncertainty represented in gray. The experimental data points are represented in black (•) and each point represents mean ± SEM. The analytical curve was compared to experimental data and the performance metrics Root-mean-square error (RMSE) and MAPE are depicted in each graphic.

In Figure 4, a representative protein from each mechanotransduction module is presented: structural (talin1, Figure 4a), intermediate ( α -actinin, Figure 4b), and signaling (FAK, Figure 4c). The model’s predictions are compared with experimental data, and the observed agreement indicates that the model accurately represents protein dynamics. The dynamic parameters $k_{In}$ and $k_{\mathrm{Out}}$ for each of the 12 FA proteins are summarized in Table 3; Supplementary Figures S2–S4.

Although the model does not explicitly calculate protein interactions, the dynamic parameters $k_{In}$ and $k_{\mathrm{Out}}$ reflect protein behavior within the FA over time. A high value of $k_{In}$ indicates frequent protein entry into the FA, while a low value suggests infrequent entry. Similarly, high $k_{\mathrm{Out}}$ values indicate more frequent protein exit from the FA, and low values suggest less frequent exit. These rates, expressed in molecules per second, represent the average behavior of the protein population. The mathematical formulations for these parameters are provided in Section 2.3.2, in Equations 2, 3.

The dynamic curves derived from these parameters align with the FRAP experimental data reported by (Stutchbury et al., 2017) (see Supplementary Figures S2–S4). In conclusion, the dynamic parameters $k_{In}$ and $k_{\mathrm{Out}}$ effectively describe FA protein dynamics in accordance with experimental observations.

#### 3.2.1 Exploring the influence of $k_{In}$ and $k_{\mathrm{Out}}$ on the dynamics FA proteins

**FIGURE 5:**
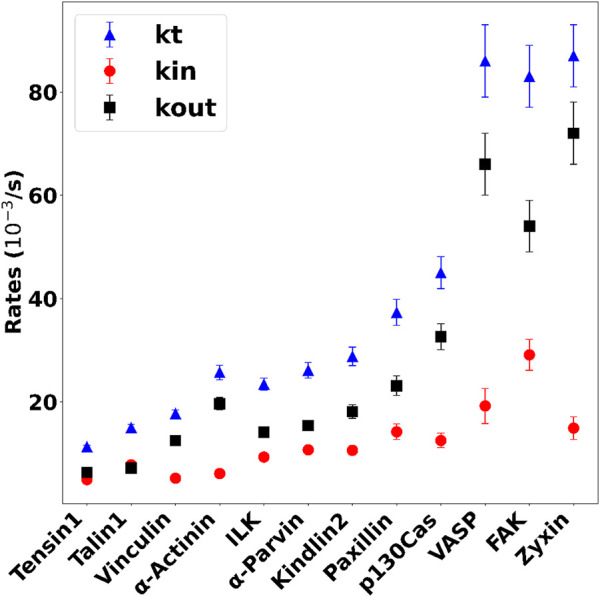
Plot of the normalized rates of all FA proteins belonging to different modules. Blue triangles are the turnover rate $\left(k_{T}\right)$ calculated from the experimental data (Stutchbury et al., 2017), black squares are the entrance rate $\left(k_{In}\right)$ and the red circles are the exit rates $\left(k_{\mathrm{Out}}\right)$ of the ROI (FA) derived from our model.

To elucidate the impact of dynamic parameters, $k_{In}$ and $k_{\mathrm{Out}}$ , in the FA proteins, we plotted the rates $k_{T}$ , $k_{In}$ and $k_{\mathrm{Out}}$ (/s) observed among all 12 FA proteins (Figure 5). Our analysis of $k_{T}$ showed consistency with the patterns previously observed experimentally by Stutchbury et al. (2017), where the proteins were subdivided into three modules: structural, intermediate, and signaling. By examining the dynamic parameters $k_{In}$ and $k_{\mathrm{Out}}$ , it is possible to verify the influence of each of these parameters in the system’s dynamics. For instance, proteins such as tensin1, talin1, ILK, α -parvin showed $k_{In}≃k_{\mathrm{Out}}$ (indicating that the rate of protein entrance is similar to the rate of protein exit), but their classification into different modules was evident when considering $k_{T}$ (turnover rate). Comparing the results obtained from our analysis directly involving $k_{In}$ and $k_{\mathrm{Out}}$ with the modules observed only using $k_{T}$ it becomes evident that $k_{In}$ and $k_{\mathrm{Out}}$ have introduced a diverse array of new dynamical behaviors. These findings underscore the significance of considering finer details of parameter dynamics to better understand biological behavior.

### 3.3 Explore dynamic interaction between proteins using $k_{In}$ and $k_{\mathrm{Out}}$

To assess the ability of the analytical solution to describe protein-protein interactions, talin was selected as a model. Talin plays a central role in cell adhesion by linking integrin receptors to the actin cytoskeleton (Figure 1). It is a large protein with a modular domain organization that contributes to its structural flexibility and diverse functions (Critchley, 2009; Gingras et al., 2009; Anthis et al., 2009; Calderwood et al., 1999; Atherton et al., 2015). In addition, talin binds to vinculin via multiple vinculin-binding sites in the talin rod region (Zhang et al., 2008; Wang, 2012; Ruoslahti, 1991). This interaction stabilizes FA and is a process that is thought to be regulated by mechanical force through talin’s interaction with integrins at the N-terminal FERM region and with F-actin at its C-terminal ABS2 and ABS3 regions (Yao et al., 2016; Chorev et al., 2018). To facilitate the understanding of the contribution of each domain of talin, the protein was divided into functional sites as shown in Supplementary Figure S5.

**FIGURE 6:**
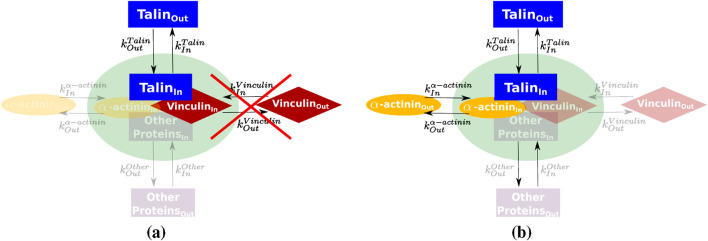
Abstract model for the network of interactions of vinculin and α -actinin with talin. **(a)** Deletion of vinculin interaction with talin. **(b)** Addition of actomyosin interaction with talin.

The network representing the interactions of talin with vinculin and α -actinin within the FA was constructed by using the individual values of $k_{In}$ and $k_{\mathrm{Out}}$ of each protein (Figure 6). The dynamics of actin are complex and involve multiple subpopulations of actin filaments with different kinetics or behaviors. This complexity makes interpreting FRAP or FLAP data for actin challenging, as these techniques assume a homogeneous population of molecules (Lippincott-Schwartz and Patterson, 2008; Watanabe and Mitchison, 2002). In this way, α -actinin, an actin-binding protein, was used as a surrogate for actin behavior as it follows the trajectory of actin (Carisey et al., 2013). We present it as a model to study the interaction of talin inside the FA. Talin full-length (FL) was used as a reference, and the dynamic rates were calculated from FLAP experiments (Supplementary Figure S6). The choice of using FLAP data in contrast to FRAP data was made because the positive fluorescence signal after photoactivation was less variable and more sensitive than the data provided by FRAP (Stutchbury et al., 2017).

To evaluate whether our stochastic framework can capture the regulatory influence of vinculin on talin dynamics (Figure 6a), we define a modified differential equation that incorporates the effect of vinculin into the dynamics of talin, as introduced in Section 2.3.3. The master equation describing the time evolution of the probability distribution of talin molecules in the bound state is given by:

$$\frac{dp\left(n_{In}^{\mathrm{Talin}},t\right)}{dt}=k_{In}^{\mathrm{Talin}}{\cdot \;n}_{\mathrm{Out}}^{\mathrm{Talin}}-k_{\mathrm{Out}}^{\mathrm{Talin}}\cdot \;n_{In}^{\mathrm{Talin}}-k_{In}^{\mathrm{V inculin}}{\cdot \;n}_{In}^{\mathrm{Talin}}$$ (10)

**FIGURE 7:**
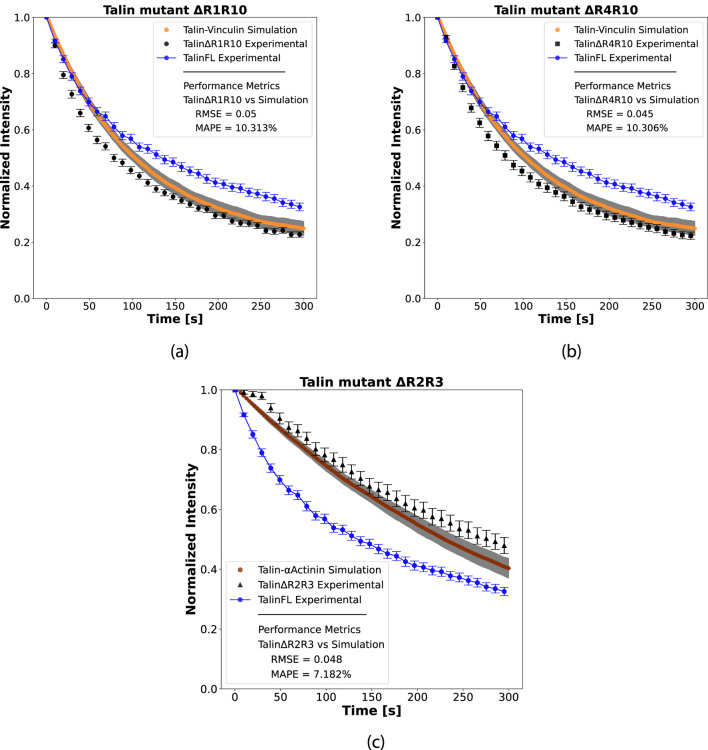
Numerical solution of the stochastic model taking into account the influence of vinculin and actin in talin dynamics. The dynamic of talin-FL at FA is represented in blue (−), which was obtained by FLAP experimental data and chemical master equation solution. The exclusion of vinculin **(a,b)** contribution resulted in an increased turnover rate of the talin dynamics (orange line represents the mean curve of the 1000 simulations, and the grey area represents the standard error). It is similar to FLAP experimental data of **(a)** talin Δ R1R10 (•) and **(b)** talin Δ R4R10 (■) , which have 9 or 4 vinculin binding sites deleted from talin, respectively. Each point of the experimental data represents mean ± SEM. **(c)** The solution of the stochastic model representing talin interacting with actin shows an increased retention of talin at FA (the amber line represents the mean curve of 1000 simulations and the grey area represents the standard error). It reproduces the FLAP experimental data of talin Δ R2R3 (▴) where the removal of talin R2R3 activates ABS2.

The term $k_{\text{In}}^{\text{Talin}}\cdot n_{\text{Out}}^{\text{Talin}}$ accounts for the transition of talin molecules from the unbound to the bound state within the FA. The second term $k_{\text{Out}}^{\text{Talin}}\cdot n_{\text{In}}^{\text{Talin}}$ describes the unbinding process of talin inside FA. The term, $k_{\text{In}}^{\text{Vinculin}}\cdot n_{\text{In}}^{\text{Talin}}$ , introduces the effect of vinculin on talin dynamics. If the rate of entrance of vinculin within FA is impaired, it will directly influence talin dynamics, which is proportional to current vinculin abundance. This formulation extends the stochastic model to incorporate molecular interaction and provides a basis for quantifying the influence of vinculin on the temporal distribution of talin states. The solution of Equation 10 considering the exclusion of the interaction of vinculin with talin, demonstrated that the contribution of the term $k_{In}^{\mathrm{V inculin}}\;{\cdot \;n}_{In}^{\mathrm{Talin}}$ is to increase the probability of talin to stay outside the FAs (Figure 7). Indeed, FLAP experimental data using talin mutants with 4 (talin Δ R4R10) or 9 (talin Δ R1R10) deleted vinculin binding sites (VBSs) have increased turnover rates when compared with talinFL (Atherton et al., 2015) (Figures 7a,b).

To incorporate the regulatory influence of actomyosin on talin dynamics, we extend the differential equation to:

$$\frac{dp\left(n_{In}^{\mathrm{Talin}},t\right)}{dt}=k_{In}^{\mathrm{Talin}}{\cdot ,\;n}_{\mathrm{Out}}^{\mathrm{Talin}}-k_{\mathrm{Out}}^{\mathrm{Talin}}\;\cdot n_{In}^{\mathrm{Talin}}+k_{In}^{\alpha -actinin}\cdot n_{In}^{\mathrm{Talin}}$$ (11)

The term $k_{\text{In}}^{\alpha -\text{actinin}}\cdot n_{\text{In}}^{\text{Talin}}$ introduces the influence of actomyosin (modeled here through the parameter associated with α -actinin) on talin behaviour. This term extends the stochastic model to incorporate cytoskeletal contributions and provides a framework for quantifying the impact of actomyosin on the temporal distribution of talin states. The solution of Equation 11 demonstrated that the interaction between actin and talin leads to increased retention of talin at FAs, which is consistent with experimental observations (Atherton et al., 2015) (Figure 7c). A previous model suggested that when talin is inactive, R2R3 remains in a closed loop bundle and requires force to change the conformation of talin to allow actin to bind to a previously masked binding region. This is in agreement with FLAP experiments where the deletion of R2 and R3 domains of talin (talin Δ R2R3) activates actin-binding site (ABS) 2 and stabilizes FA independent of vinculin (which itself has a stabilizing effect, Supplementary Figure S7).

Altogether, our data demonstrate that our model can be extended to infer protein interactions. By using the value of the dynamic rates of full-length talinFL (wild-type) and modifying its interactions with vinculin and actomyosin, we effectively simulated scenarios representing a loss of vinculin interaction (resulting in increased talin turnover rate), and constitutive binding to actomyosin (resulting in reduced talin turnover).

## 4 Discussion

We developed a stochastic mathematical model based on the analytical solution of the chemical master equation to infer dynamic rates, $k_{In}$ and $k_{\mathrm{Out}}$ , from FRAP and FLAP experiments of FA proteins (Table 3; Supplementary Table S2). These rates provide additional information on the dynamic behavior of proteins beyond the turnover rate, $k_{T}$ , typically extracted from experimental data. Furthermore, our model was cross-validated using *K-Fold* cross-validation technique eliminating the possibility of overfitting (Table 2).

The model describes different protein dynamics with the same accuracy as determined experimentally, distinguishing between proteins in the mechanotransduction modules as previously proposed (Stutchbury et al., 2017): structural (tensin1, talin1, and vinculin, Supplementary Figure S2), intermediate ( α -actinin, ILK, α -parvin, kindlin2, Supplementary Figure S3), and signaling (paxillin, p130Cas, VASP, FAK and zyxin, Supplementary Figure S4). While previous studies attempted to extract dynamic parameters from FRAP and FLAP experiments (Sprague et al., 2004; Sprague and McNally, 2005; Alexander and Lawley, 2022; Kang et al., 2009; Kang, 2020) our model is the first to use the formalism of the chemical master equation to study the dynamic behavior of FA proteins. Additionally, with only two parameters, $k_{In}$ and $k_{\mathrm{Out}}$ our model effectively captures the phase transition of protein dynamic, from a slower mechanical interaction to a faster biochemical interaction (Supplementary Figures S2–S4; Table 3). Each FA protein exhibits unique mechanisms in its dynamics, and our stochastic model can describe them all based on the values of $k_{In}$ and $k_{\mathrm{Out}}$ . It also provides new insights into how the proteins interact and behave in the FA. Examination of $k_{In}$ and $k_{\mathrm{Out}}$ (Table 3; Figure 5) revealed that $k_{\mathrm{Out}}$ predominantly influences the protein dynamics and represents how the proteins interact within FAs. Low values of $k_{\mathrm{Out}}$ indicate strong interactions, while high values indicate weak interactions within FAs. $k_{In}$ showed minimal variation between proteins and represented the protein behavior outside of FAs. Note that proteins from the structural module, such as tensin, talin and vinculin, interact directly with integrins and have a smaller value of $k_{In}$ and $k_{\mathrm{Out}}$ . In contrast, proteins from the signaling module show higher values of $k_{In}$ and $k_{\mathrm{Out}}$ . This trend is evident in the behaviors of tensin and FAK, as illustrated in Figure 5.

Several models have been described in the literature to extract dynamic parameters from FRAP and FLAP experiments. The reaction-diffusion model integrates molecular diffusion processes and chemical reactions (such as binding and unbinding) to represent molecular movement and interactions within cells (Carrero et al., 2003; Phair and Misteli, 2001; Sprague et al., 2004; Mueller et al., 2008). Key parameters inferred from FRAP experiments using this model include the diffusion coefficient, binding rates, and unbinding rates. However, a significant challenge is to accurately estimate these parameters when the underlying model assumptions are oversimplified, potentially resulting in misleading interpretations and compromising the predictive reliability of the model (Mai et al., 2011). On the other hand, kinetic models focus on biochemical reaction rates, such as binding and unbinding, and use ordinary differential equations (ODEs) to describe the temporal evolution of molecular concentrations (Mai et al., 2011; Sprague et al., 2004). It is a deterministic model that may not fully capture the stochastic nature of biological systems. For more realistic representations, stochastic models such as Monte Carlo simulations can apply random sampling to probabilistically model molecular movements and interactions in FRAP experiments (Mueller et al., 2008).

However, these simulations are computationally demanding, and require substantial computational resources and time, particularly for complicated or large-scale models (Lorén et al., 2015). Another alternative for parameter estimation is the Hidden Markov Model (HMM), which uses probabilistic frameworks to describe systems where the state (hidden) is inferred from observable data (emissions). In FRAP experiments, HMMs can model the temporal dynamics of molecular interactions through transitions between hidden states governed by transition and emission probabilities (Braeckmans et al., 2003; Braga et al., 2004; Mazza et al., 2008). However, the challenges of this type of modeling include the complex estimation of these probabilities, especially in the presence of noisy data or numerous hidden states. In the present work, we present a straightforward model using the chemical master equation and two parameters traditionally obtained from FRAP and FLAP experiments: the turnover rate $k_{T}$ and the stationary concentration of mobile proteins $\left(\bar{n_{in}^{P}}\right)$ . Unlike the other proposed mathematical models, it does not require any prior assumptions about the proteins, takes into account the stochastic nature of the experiments, and provides an exact analytical solution for the system. The limitation of this approach is the assumption that the molecules of the system are uniformly distributed within the reaction volume since the cellular environment is spatially heterogeneous or compartmentalized. Yamashiro et al. (2023) (Yamashiro et al., 2023), used single-molecule imaging studies to show that focal adhesion proteins, such as talin, exist in multiple binding states: bound only to actin filaments, attached only to integrins, or bridging both actin and integrins. This heterogeneity is a key feature of focal adhesion dynamics and contributes to the regulation of cytoskeletal organization and cellular signaling. The ability of single-molecule approaches to distinguish between these states, particularly through the tracking of retrograde actin flow, provides mechanistic insights that are not directly accessible through ensemble techniques such as FRAP or FLAP. However, while FRAP/FLAP inherently averages the behavior of a mixed population of molecules, our modeling framework incorporates the dynamics of protein interactions via the kinetic parameters $k_{In}$ and $k_{\mathrm{Out}}$ . These parameters allow us to resolve subpopulations within the mobile fraction based on their distinct exchange kinetics. By integrating interaction-based dynamics, our approach provides a potential means to infer heterogeneous behaviors within focal adhesion complexes, complementing and extending the interpretive power of conventional fluorescence recovery methods.

The chemical master equation also efficiently describes protein-protein interactions and how they affect protein dynamics. This information cannot be obtained from experimental data alone (Sprague and McNally, 2005; Lippincott-Schwartz et al., 2018; Geverts et al., 2015; Williamson et al., 2021). Knowing the values of $k_{In}$ and $k_{\mathrm{Out}}$ for each protein, enabled to predict the influence of vinculin and actin interaction on talin dynamics (Figures 6, 7). The increased probability of talin remaining outside the FAs consistent upon elimination of vinculin interaction is consistent with FLAP experiments showing increased turnover of talin mutants with deleted vinculin binding sites (Figure 7). Indeed, cells depleted of vinculin have smaller and more dynamic FAs (Atherton et al., 2015), showing that the absence of vinculin will increase the turnover rate of talin in FAs leading to less stable adhesions. On the other hand, the interaction of actin with talin results in increased retention of talin at FA (Figure 7) similar to FLAP experimental data of talin with deleted R2 and R3 domains, which was shown to result in the unmasking of the actin-binding site (ABS2) and a vinculin independent stabilization of FAs (Atherton et al., 2015).

Here, we used α -actinin data to represent actin dynamics. Whilst not ideal we believe that it best represents actin dynamics when bundled and in connection with the adhesion plaque. The reason for this is the observation that in experiments with expression of constitutively active vinculin FAs, many other proteins become stabilized in FAs, resisting even cytochalasin D treatment. In the same cells α -actinin follow the dynamics of disrupted actin cytoskeleton, suggesting their tight association (Carisey et al., 2013). In addition, α -actinin and actin are absent from vinculin-stabilized FAs (Carisey et al., 2013), further supporting the idea that α -actinin is part of an actin regulatory module as observed by super-resolution microscopy (Kanchanawong et al., 2010; Liu et al., 2015). Our data show that although α -actinin has an intermediate value of $k_{T}$ , the dynamics of $k_{In}$ and $k_{\mathrm{Out}}$ are more similar to the signaling proteins. It deserves further investigation to define better and characterize the proteins belonging to this new actin regulatory module.

**FIGURE 8:**
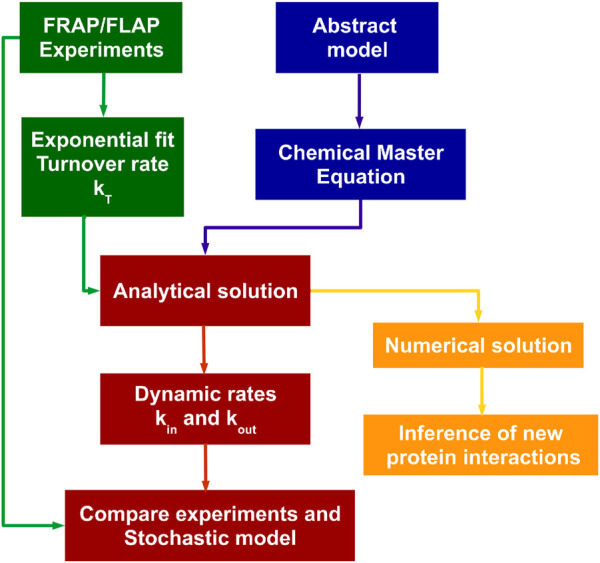
Strategy workflow of the analytical solution of the chemical master equation to infer dynamic rates from FRAP and FLAP experiments. The experimental data (green boxes) allow the extraction of turnover rate $\left(k_{T}\right)$ and the stationary distribution of the protein $\left(\bar{n^{P}}\right)$ is equivalent to fluorescence intensity. The analytical solution (red boxes) utilizes the stochastic mathematical model represented by the chemical master equation (blue boxes) combined with experimental data (green boxes) to extract the dynamic rates $k_{In}$ and $k_{\mathrm{out}}$ to obtain the temporal evolution of the protein behavior which is compared to experimental data. The values of $k_{In}$ for individual proteins allow the prediction of protein-protein interaction by the numerical solution (yellow) using the chemical master equation.

In summary, our model navigates in various environments and can describe very different protein dynamics. Furthermore, it predicts information for the influence of protein interaction using only $k_{In}$ and $k_{\mathrm{Out}}$ of the interacting proteins. The prediction was validated using the information from the full-length wild-type proteins and the mutants that affect the protein interaction. We employed the analytical solution using the chemical master equation to extract new dynamic parameters and introduce a more refined observation of FA protein behavior than $k_{T}$ alone. Importantly, our model can be extended to study any other proteins of interest as illustrated in the schematic workflow presented in Figure 8. Our findings highlight the analytical solution as a valuable tool for conducting *in silico* testing of protein interactions, thereby offering new experimental insights into FA dynamics and various other cellular processes.

## Data Availability

The original contributions presented in the study are included in the article/Supplementary Material, further inquiries can be directed to the corresponding authors.
