# Supplementary material for: Exploring focal adhesion data: dynamic parameter extraction from FRAP and FLAP experiments using chemical master equation
Source: Front Mol Biosci. 2025 May 6;12:1587608. doi: 10.3389/fmolb.2025.1587608 (PMC12088951; doi:10.3389/fmolb.2025.1587608)
Supplement: Supplementary file 1 [file DataSheet1.pdf]

Supplementary Material

1 SUPPLEMENTARY MATERIAL

1.1 Extracting dynamic parameters from FRAP and FLAP experiments

FRAP and FLAP experimental data consist of fluorescence intensity curves representing the recovery or the loss of intensity, respectively. These curves have an exponential shape and can be fitted using the equation $y = \ y_0 + Ae^{R_0 x}$, thus yielding values for the parameters y_0, A, and R_0. Here we have the representation of a recovery curve from a FRAP experiment (Figure S1) that we used to recover the value of the turnover rate $k_T = |R_0|$. The stationary concentration of proteins $(\overline{n_{in}^P})$, is inferred based on the fluorophore intensity of the last time point of the experiment, that represent the population of the protein in the R.O.I.

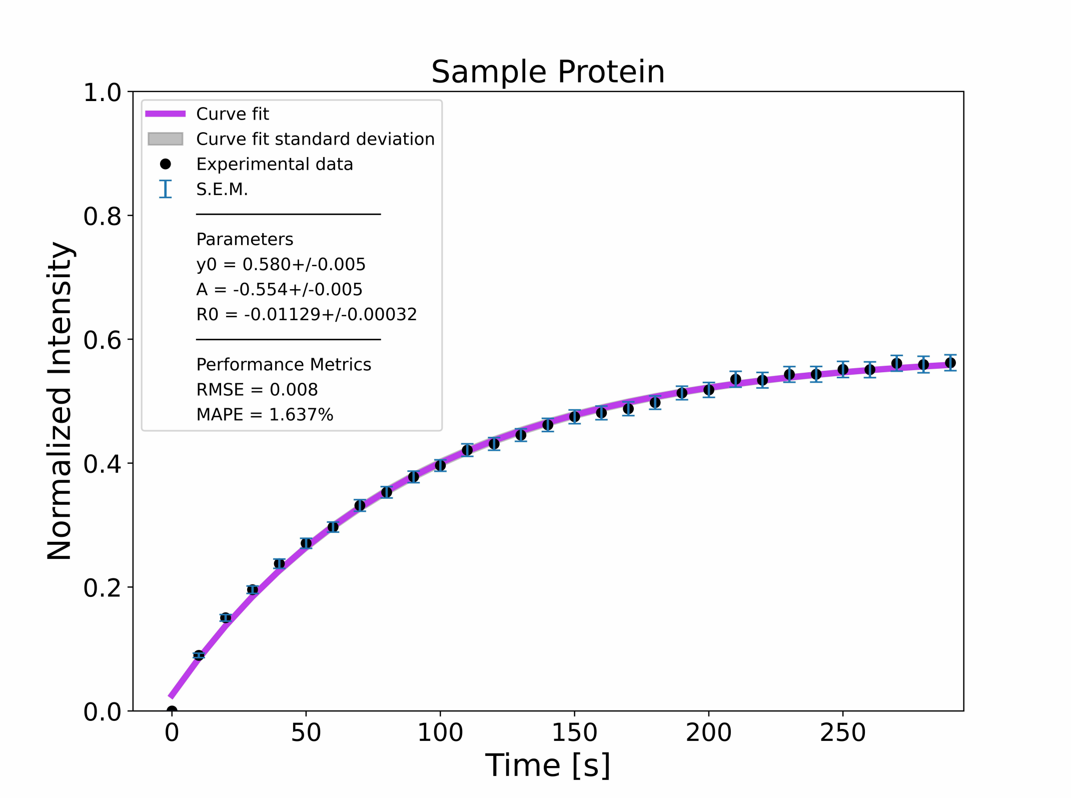

Figure S1: FRAP experimental data fitted by the equation $y = \ y_0 + Ae^{R_0 x}$. $|R_0|$ represents the mobility of the protein, or the turnover rate k_T.

1.2 Parameters for the analytical solution of individual proteins

From equation (3) in the main text,

$$\begin{aligned} n_{In}^{P}(t) = n_{In}^{P}(t_0)\frac{k_{Out}^{Protein} + k_{In}^{Protein} exp(-(k_{Out}^{Protein} + k_{In}^{Protein})(t - t_0))}{k_{Out}^{Protein} + k_{In}^{Protein}} \\ + n_{Out}^{P}(t_0)\frac{k_{Out}^{Protein}(1 - exp(-(k_{Out}^{Protein} + k_{In}^{Protein})(t - t_0)))}{k_{Out}^{Protein} + k_{In}^{Protein}} \end{aligned} \tag{S1}$$

we have the following unknown parameters:

- $n_{In}^{P}(t_0)$ initial concentration of proteins inside the Focal Adhesion (FA).
- $n_{Out}^{P}(t_0)$ initial concentration of proteins outside the FA.
- $k_{In}^{Protein}$ rate of each protein going inside the FA.
- $k_{Out}^{Protein}$ rate of each protein going outside the FA.
- t total time of the experiment.
- t_0 initial time of the experiment.

The experimental data does not provide the absolute population values of the proteins; instead, we only have the relative fluorescence values for each protein. For that reason, it was assumed that the initial concentration of proteins to be $n_{In}^{P}(t_0) = 0$ molecules, and the population outside the focal adhesion to be $n_{Out}^{P}(t_0) = 1000$ molecules for all proteins. The initial time $t = 0s$ and total time of the experiment is set to $t = 300s$. The results are normalized to the maximum number of proteins to facilitate comparison with the normalized fluorophore intensity obtained from FRAP experiments.

The values of k_{In} and k_{Out} of each FA protein were calculated using equation (6, main text). We used these values in Table 3, main text, to calculate the time evolution of each protein and compare with experimental data (Figures 5 and 8 in the main text and supplementary Figures S2, S3 and S4).

1.3 Calculation of dynamic parameters for the analytical solution of the chemical master equation

Since the model was validated and proved to be able to describe experimental data, we used the whole set of experiments to extract k_T and n_{In}^P and used it to calculate the dynamic parameters k_{In} and k_{Out}. We are presenting here the individual plots of the analytical model for the different proteins. The number of experiments used for each protein matches the column "Final number of experiments" presented in Table S1.

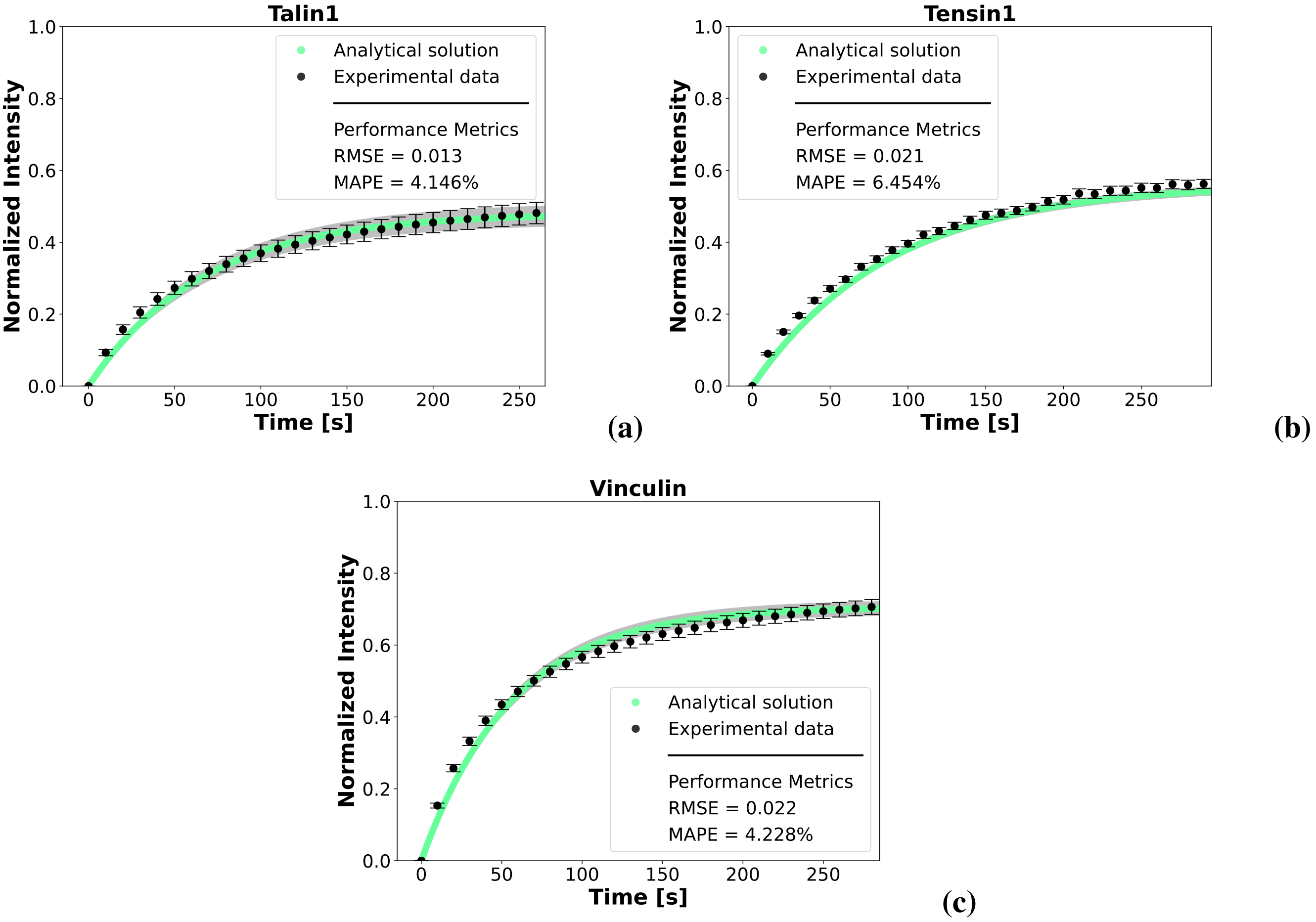

Figure S2: Mecanosensing module proteins: (a) Talin1 (N=32); (b) Tensin1 (N=78); (c) Vinculin (N=32). Analytical curves in green (•) are compared to experimental data points represented in black (•). The analytical curve uncertainty is represented in gray, while each point of experimental data represents the mean ± SEM. The goodness of fit metrics RMSE and MAPE are presented in each graphic legend and in Table 3.

1.4 Talin

To facilitate the understanding of the contribution of each domain of talin, the protein was divided into functional sites as shown in Figure S5. Additionally TalinFL FLAP experiment was modeled in the same way as the before mentioned FRAP experiments (Sections 2.1 and 2.2 main text and Section 1.2 supplementary material), as shown in Figure S6.

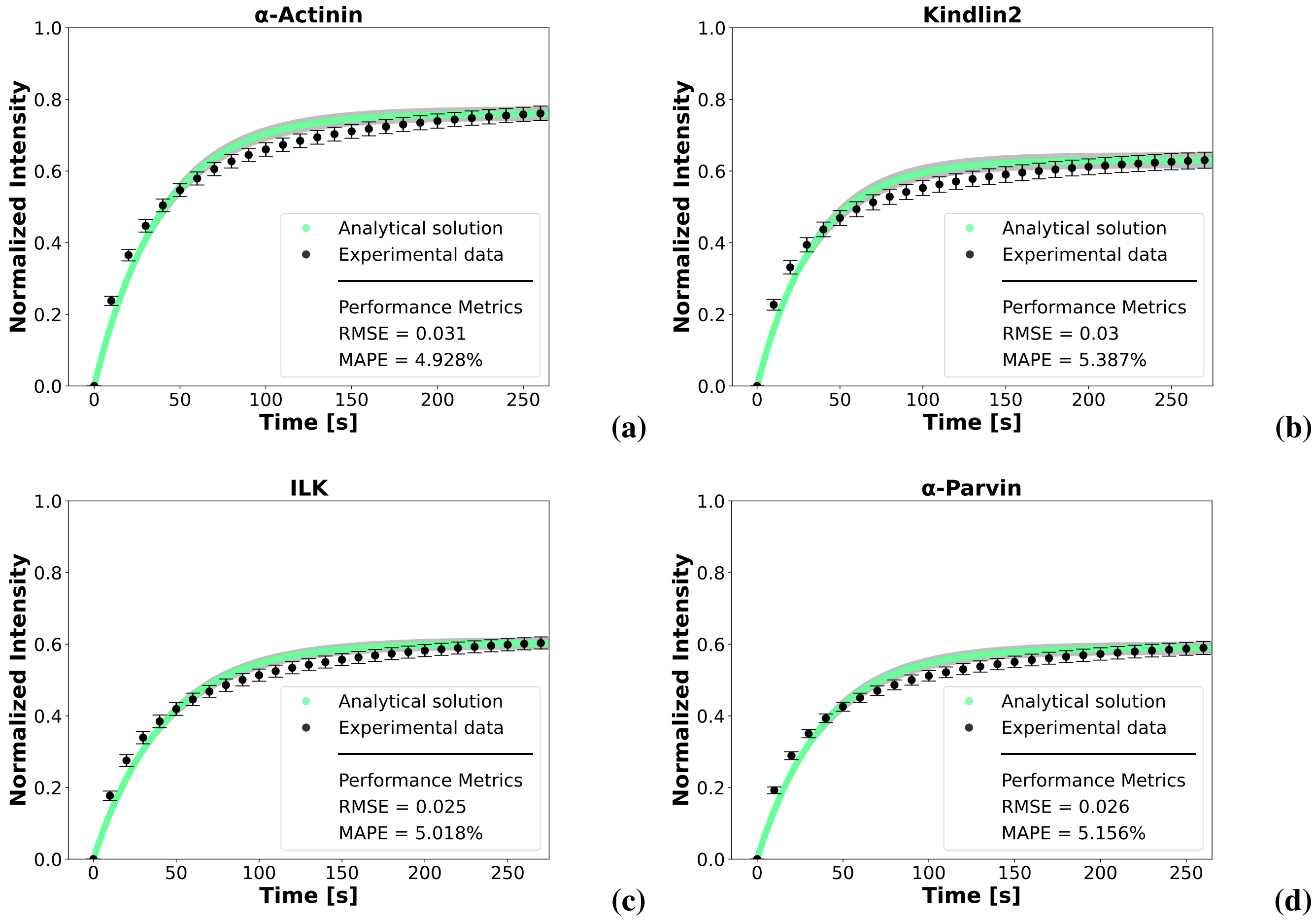

Figure S3: Intermediate module proteins: (a) α-Actinin (N=55); (b) Kindlin2 (N=49); (c) ILK (N=37); (d) α-Parvin (N=48). Analytical curves in green (•) are compared to experimental data points represented in black (•). The analytical curve uncertainty is represented in gray, whileeach point of experimental data represents the mean $\pm$ SEM. The goodness of fit metrics RMSE and MAPE are presented in each graphic legend and in Table 3.

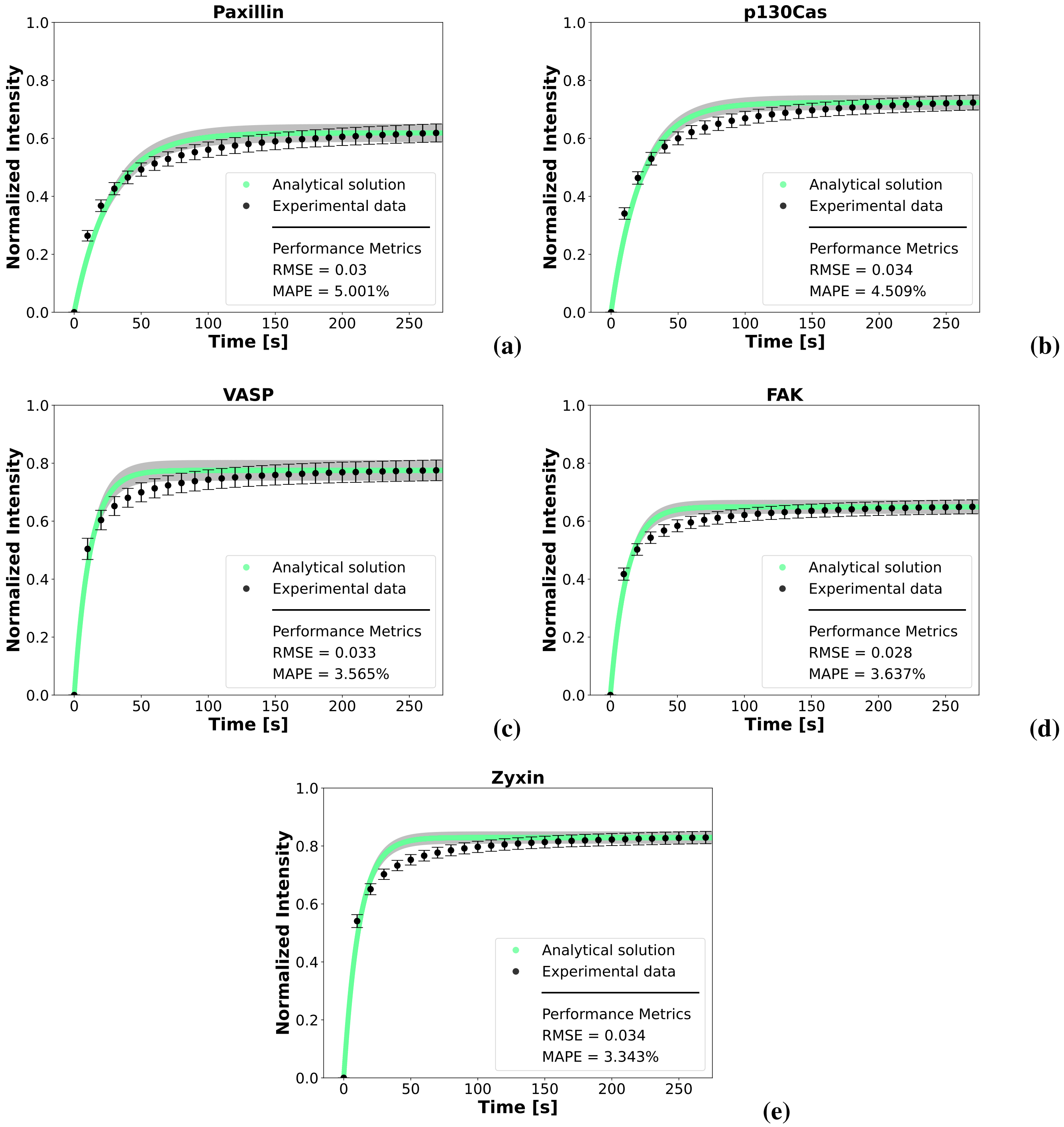

Figure S4: Mecanosignaling module proteins: (a) Paxillin (N=24); (b) p130-Cas (N=32); (c) VASP (N=24); (d)FAK (N=35); (e) Zyxin (N=34).Analytical curves in green (•) are compared to experimental data points represented in black (•). The analytical curve uncertainty is represented in gray, while each point of experimental data represents the mean ± SEM. The goodness of fit metrics RMSE and MAPE are presented in each graphic legend and in Table 3.

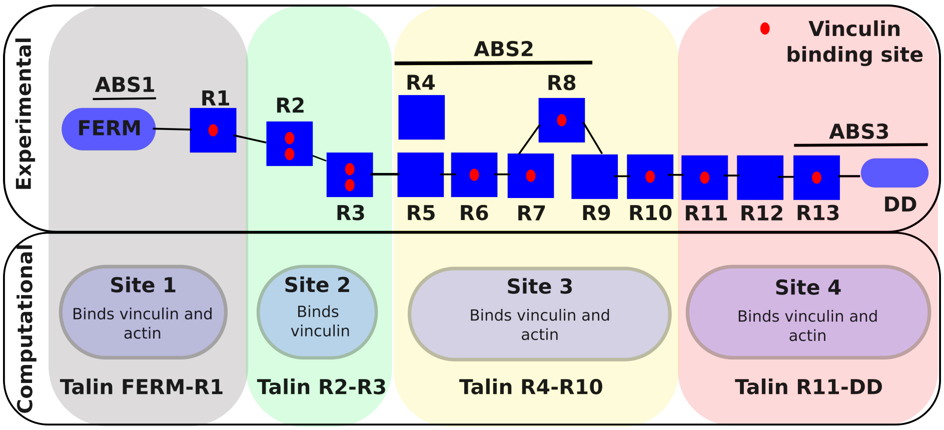

Figure S5: Schematic representation of functional domains of talin considered in experimental (above) and computational (below) approaches. Structurally, talin has 11 potential vinculin binding sites (red dots) and 3 actin-binding sites (ABS1, ABS2, and ABS3). To facilitate the understanding of the contribution of each domain of talin, the protein was divided into functional sites: site 1: FERM and R1 domains, site 2: R2-R3 domains, site 3: R4-R10 domains, site 4: R11-DD. Sites 1, 3, and 4 bind vinculin and actin, and site 2 binds only vinculin. These sites were taken into account to validate the prediction of interaction of talin with vinculin and actin using experimental mutations and deletions performed in the talin.

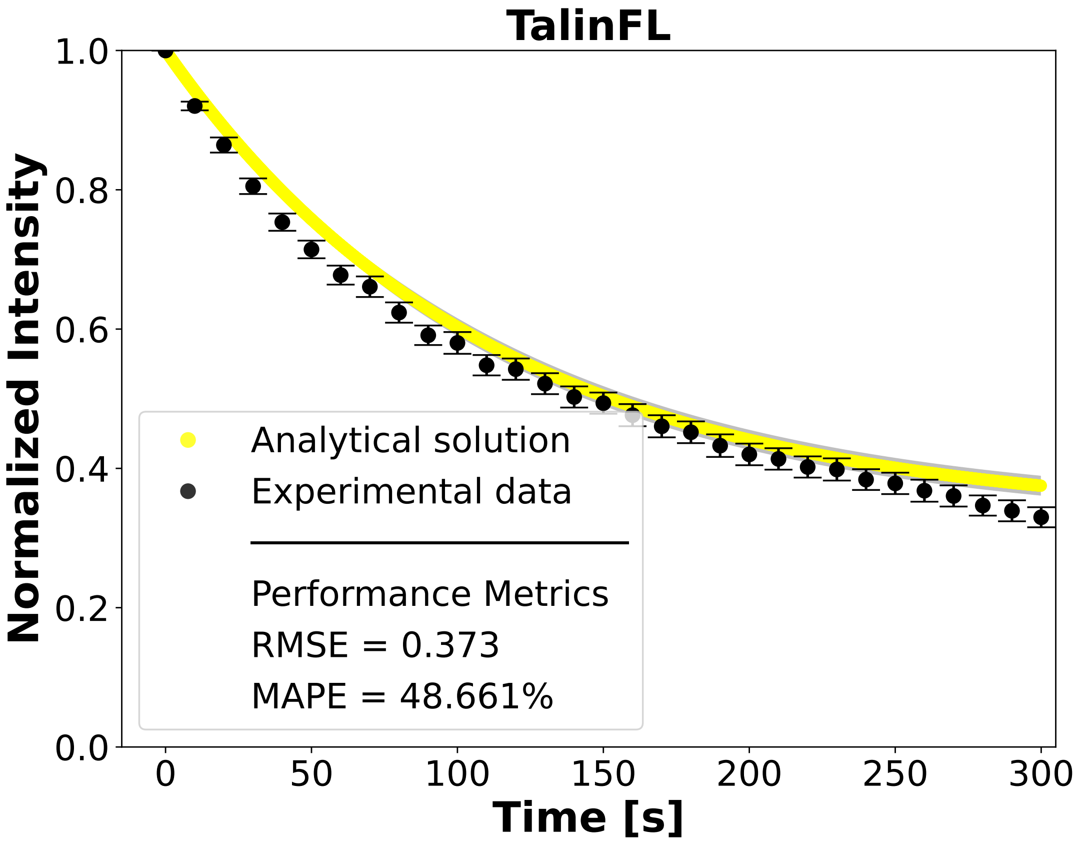

Figure S6: Analytical curve of talinFL, obtained from FLAP experiment, modeled using the same equations for FRAP experiments (Sections 2.1 and 2.2 main text and Section 1.2 supplementary material).. The analytical curve is represented in yellow(•) with the uncertainty represented in gray. The experimental FLAP data points are represented in black(•) with each point of experimental data representing the mean ± SEM. The analytical curve was compared to experimental data and the performance metrics RMSE and MAPE are depicted in the graphic. The calculated dynamic parameters for this curve are presented in Table S2

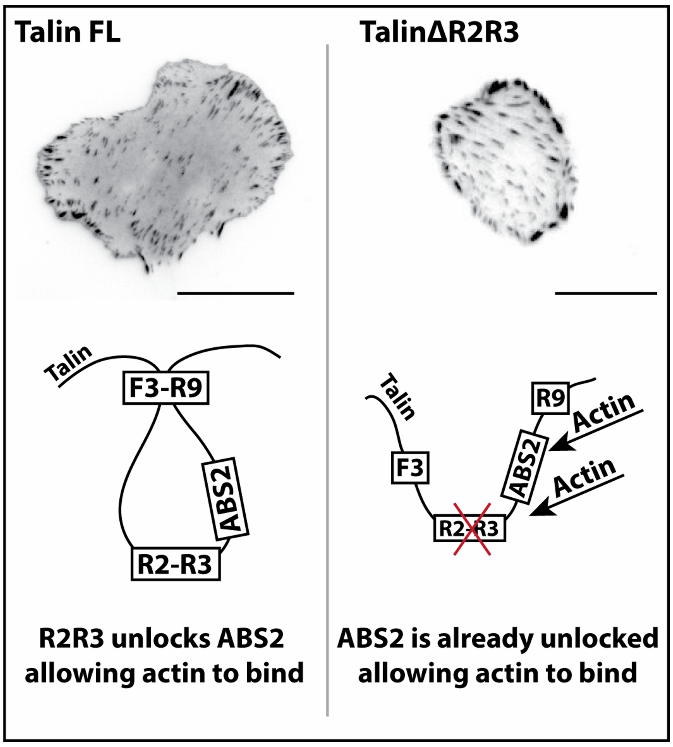

Figure S7: When talin-FL is inactive, R2-R3 domain remains in a closed loop bundle and requires forces to unravel talin to allow actin to bind. The absence of these domains talinΔR2R3, eliminates this requirement for force-dependent unravelling, thereby constitutively unlocking ABS2, leading to actin binding. Therefore, talinΔR2R3 form larger FAs than those seen with talinFL and remains stable even in the presence of actomyosin-inhibiting reagents. The increased stability of talinΔR2R3 is most likely due to the availability of actin-binding at ABS2.

1.5 Supplementary Tables

Table S1. Number of experimental data available to analysis

Protein	Technique	Experiments (curves)	Outliers	Final experiments
**α-Actinin**	FRAP	56	1	55
**α-Parvin**	FRAP	53	5	48
**FAK**	FRAP	35	0	35
**ILK**	FRAP	39	2	37
**Kindlin2**	FRAP	57	8	49
**p130Cas**	FRAP	38	6	32
**Paxillin**	FRAP	26	2	24
**Talin1**	FRAP	32	0	32
**Tensin1**	FRAP	86	8	78
**VASP**	FRAP	24	0	24
**Vinculin**	FRAP	35	3	32
**Zyxin**	FRAP	34	0	34
**TalinFL**	FLAP	66	4	62
**TalinΔR4R10**	FLAP	72	2	70
**TalinΔR1R10**	FLAP	62	2	60
**TalinΔR2R3**	FLAP	51	2	49

Table S2. Distribution of dynamic parameters for the different talin proteins.

Protein	k_T (10^{-3}/s)	k_{In} (10^{-3}/s)	k_{Out} (10^{-3}/s)	$\overline{n_{out}^P}$ (Normalized Intensity)
**TalinFL**	8.98±0.35	2.96±0.17	6.02±0.27	0.330±0.014
**dltR4-R10**	11.62±0.31	2.64±0.17	8.98±0.28	0.227±0.013
**dltR1-R10**	11.7±0.4	2.67±0.15	9.03±0.35	0.228±0.009
**dltR2R3**	2.87±0.34	1.39±0.18	1.48±0.19	0.484±0.028

Table S3. *K-Fold* cross-validation with outliers results: The results are interpreted following Table 1. For the three studied values of K(3, 5 and 10), 100 reproductions were performed, the averages of MAPE values are presented with their respective uncertainties.

	K = 3		K=5		K=10	
**Protein**	**MAPE(%)**	**Interpretation**	**MAPE(%)**	**Interpretation**	**MAPE(%)**	**Interpretation**
**Tensin1**	6.37+/-0.12	Highly accurate	6.86+/-0.11	Highly accurate	8.06+/-0.13	Highly accurate
**Talin1**	11.5+/-0.4	Good	13.2+/-0.4	Good	17.9+/-0.4	Good
**Vinculin**	6.82+/-0.18	Highly accurate	7.43+/-0.15	Highly accurate	8.93+/-0.15	Highly accurate
**α-Actinin**	7.07+/-0.18	Highly accurate	7.55+/-0.14	Highly accurate	9.28+/-0.13	Highly accurate
**ILK**	9.04+/-0.24	Highly accurate	9.08+/-0.21	Highly accurate	11.36+/-0.21	Good
**α-Parvin**	7.67+/-0.19	Highly accurate	8.30+/-0.17	Highly accurate	9.93+/-0.15	Highly accurate
**Kindlin2**	8.73+/-0.25	Highly accurate	9.98+/-0.20	Highly accurate	11.81+/-0.17	Good
**Paxillin**	10.37+/-0.31	Good	11.94+/-0.29	Good	15.9+/-0.4	Good
**p130Cas**	7.82+/-0.23	Highly accurate	8.99+/-0.19	Highly accurate	11.34+/-0.20	Good
**VASP**	8.79+/-0.33	Highly accurate	11.06+/-0.34	Good	14.62+/-0.27	Good
**FAK**	6.98+/-0.24	Highly accurate	8.10+/-0.20	Highly accurate	10.93+/-0.20	Good
**Zyxin**	5.37+/-0.14	Highly accurate	6.22+/-0.12	Highly accurate	7.59+/-0.11	Highly accurate
